# Genetic Profiles of Ten African Swine Fever Virus Strains from Outbreaks in Select Provinces of Luzon, Visayas, and Mindanao, Philippines, Between 2021 and 2023

**DOI:** 10.3390/v17040588

**Published:** 2025-04-21

**Authors:** Andrew D. Montecillo, Zyne K. Baybay, Jimwel Bryan Christopher Ferrer, Wreahlen Cariaso, Airish Pantua, John Paulo Jose, Rachel Madera, Jishu Shi, Karla Cristine Doysabas, Alan Dargantes, Kassey Alsylle T. Dargantes, Anna Rochelle A. Boongaling, Alfredo P. Manglicmot, Lucille C. Villegas, Homer D. Pantua

**Affiliations:** 1 BioAssets Corporation, City of Santo Tomas 4234, Batangas, Philippines; zyne.baybay@bioassets.com.ph (Z.K.B.); jimwel.ferrer@bioassets.com.ph (J.B.C.F.); wreah.cariaso@bioassets.com.ph (W.C.); airish.gatlabayan@bioasssets.com.ph (A.P.); 2 Institute of Biological Sciences, University of the Philippines Los Baños, Laguna 4031, Philippines; lcvillegas1@up.edu.ph; 3 Industrial Technology Development Institute, Department of Science and Technology (DOST-ITDI), Bicutan, Taguig City 1631, Philippines; jpgjose@itdi.dost.gov.ph; 4 Center on Vaccine Evaluation and Alternatives for Antimicrobials, Kansas State University, Manhattan, KS 66506, USAjshi@vet.k-state.edu (J.S.); 5 College of Veterinary Medicine, Central Mindanao University, Maramag, Bukidnon 8710, Philippines; laladoysabas@yahoo.com (K.C.D.); alanpdargantes@gmail.com (A.D.); kingdargantes@gmail.com (K.A.T.D.); 6 Provincial Veterinary Office of Oriental Mindoro, Calapan City 5200, Oriental Mindoro, Philippines; provet_ormdo@yahoo.com (A.R.A.B.); provet.oriental.mindoro@gmail.com (A.P.M.)

**Keywords:** African swine fever virus, ASF, genomic epidemiology, whole-genome sequencing

## Abstract

An African Swine Fever (ASF) outbreak was first recorded in the Philippines in July 2019. Since then, the disease has spread across provinces in Luzon, Visayas, and Mindanao, causing severe economic consequences for the country’s swine industry. Here, we report the genome sequencing of ASF virus strains from outbreaks in several provinces of the Philippines between 2021 and 2023, using a long-read tiled amplicon sequencing approach. The coding-complete genomes generated ranged from 187,609 to 189,540 bp in length, with GC contents of 38.4% to 38.5%. Notably, a strain from the Bataan province had a 1.9 kb deletion at the 5′-end, affecting several coding regions. The strains were characterized using 13 genes and regions; namely the *B646L* gene, the CD2v serogroup, the central variable region (CVR) of the *B602L* gene, the intergenic region (IGR) between the *I73R* and *I329L* genes, the IGR between *A179L* and *A137R*, *O174L*, *K145R*, *Bt*/*Sj*, *J268L*, and *ECO2*, the multigene family (MGF) 505-5R, and the MGF 505-9R and 10R regions. The ASFV strains were mostly related to Asian and European p72 genotype II strains. Genetic profiling provides valuable information on the diversity of local strains of ASFV in the Philippines, which are useful for epidemiology, diagnostics, and vaccine development.

## 1. Introduction

Infectious diseases remain the primary cause of losses in the production of animals for food. In the Philippines, recent outbreaks of African swine fever (ASF) and bird flu have been putting pressure on the animal production sector to meet the growing demand for high-value animal protein and products. Since 2005, ASFV has caused an estimated loss of at least nine million animals globally. In the Philippines, since its first detection in 2019, at least half a million animal losses have been reported. Along with Indonesia, Malaysia, Thailand, and Vietnam, the Philippines is one of the five Asian countries affected by these diseases with emerging markets in livestock production and consumption [1].

First reported in Kenya in the 1920s, ASF is a highly lethal hemorrhagic disease of domestic and wild swine, with mortality rates approaching 100% [2]. It is caused by a large, enveloped, double-stranded DNA virus of the genus *Asfivirus*, family *Asfarviridae* with a genome size of 170 to 194 kb [3,4]. The virus replicates predominantly in monocytes and macrophages of the mononuclear phagocyte system, and in the late stages of infection other cell types may also be infected [5]. Feeding of swill or untreated food scraps to animals, and illegal movement (smuggling) of animals and pig products across boundaries are seen as the most common sources of the occurrence and rapid spread of ASF [6]. ASFV currently has a very narrow host range with no zoonotic potential, and there are no indications that this will change [7,8].

Since its re-emergence in Georgia in 2007, ASF has continued to spread across Caucasia and Eastern-Europe, reaching the Caribbean, China, and several Southeast Asian countries [9,10,11,12,13,14,15]. In the Philippines, the first outbreak was recorded in July 2019, leading to a significant reduction in the country’s swine population by around three million hogs. This has resulted in losses of more than PHP 100 billion and increases in the prices of pork products. As of April 2024, ASF has already spread to 17 administrative regions, including a total of 73 provinces [16]. A recent study in the Philippines pinpointed the primary factors fueling the spread of ASF. The findings highlighted swill feeding, lax farm safety protocols, and personnel movement as major contributors to the disease’s transmission [17]. ASF has been considered a significant threat to worldwide pork production and, currently, no therapy or approved vaccine exists against the disease [18]. However, several countries, including Spain, have successfully eradicated ASF. This was made possible by sufficient funding for the establishment of a network of control measures, the deployment of mobile veterinary teams, and the operation of a reference laboratory for ASF surveillance and outbreak detection [19].

Since the first report of ASF in the Philippines in 2019 and its rapid spread throughout the country, several genome sequences of locally circulating strains have already been published or uploaded to online repositories [20,21]. This study aimed to add to that collection of data and to characterize the genomes of the circulating strains of ASFV from outbreaks in selected provinces of the Philippines between 2021 and 2023 by using a multi-gene-based approach. Specifically, the genomes of the field strains were characterized based on the *B646L* gene, the CD2v serogroup, the central variable region (CVR) of the *B602L* gene (CVR_B602L_), the intergenic region (IGR) between the *I73R* and *I329L* genes (IGR_I73R-I329L_), the IGR between *A179L* and *A137R* (IGR_A179L-A137R_), other regions such as *O174L*, *K145R*, *Bt*/*Sj*, *J268L*, and ECO2, various *multigene families* (*MGF*) such as *MGF 505-5R*, and the *MGF 505-9R* and *10R* regions, and were compared to other related ASFV strains. The selected genetic markers were chosen for their relevance in phylogenetic classification, molecular epidemiology, and virulence profiling of ASFV as established and demonstrated previously in several studies [14,15,22,23,24]. Specifically, *B646L* (p72) is a highly conserved gene used for genotyping and lineage classification, while *EP402R* (CD2v) encodes a hemadsorption protein involved in serogrouping and host interaction. The CVR of *B602L* offers high-resolution subtyping due to its hypervariability. IGRs, particularly between *I73R* and *I329L*, accumulate single nucleotide polymorphisms and indels useful for fine-scale geographic tracking of outbreaks. Moreover, *O174L* and *K145R* are emerging markers showing region-specific polymorphisms and potential links to recombinant variants. MGFs (e.g., *MGF 360* and *MGF 505*) are associated with immune evasion and virulence, and their diversity can provide insights into strain pathogenicity and adaptation. These markers were analyzed to monitor the potential emergence of new variants among circulating strains in the Philippines and could be useful in the source tracking of strains. Genomes were analyzed for the presence of structural variants, such as long insertion or deletion, and the possible detection of recombinants. Furthermore, genomes were also compared to representative genomes of various ASFV genotypes to infer evolutionary relatedness. To our knowledge, this is the first report on the genetic characterization of genomes of ASFV strains from outbreaks in the Philippines between 2021 and 2023 generated through a portable long-read sequencing platform.

## 2. Materials and Method

### 2.1. Sample Collection and Viral DNA Isolation

Whole blood samples (*n* = 10) were collected from domestic pigs exhibiting signs and symptoms of ASF by licensed veterinary consultants. The collection followed the guidelines of the Bureau of Animal Industry, Department of Agriculture (Philippines), during reported outbreaks in several locations in the Philippines (Table 1). Samples were selected based on geographic location to include at least one sample per major island group in the country. Blood samples were stored in ice or in cool packs and sent to the BioAssets Veterinary Research and Diagnostic Laboratory (Sto. Tomas, Batangas, Philippines) for molecular diagnostics and further processing. The total DNA was extracted using the MagMax™ DNA Multi-Sample Kit (Thermo Fisher Scientific, Waltham, MA, USA) or the IndiSpin Pathogen Kit (INDICAL Bioscience, Leipzig, Germany) following the manufacturer’s protocol. The quality of the DNA extracts was assessed spectrophotometrically using the DeNovix DS-11 microdrop spectrophotomer (DeNovix, Wilmington, DE, USA) and visually confirmed by agarose gel electrophoresis. The extraction yield and quantity were measured using the Qubit™ 1x dsDNA HS Assay Kit (Thermo Fisher Scientific, Waltham, MA, USA) on a Qubit 4.0 fluorometer (Thermo Fisher Scientific, Waltham, MA, USA). The presence of the ASFV in the samples was confirmed either by quantitative PCR using the VetMax™ African Swine Fever Virus Detection Kit (Applied Biosystems, Waltham, MA, USA) or the Indical virotype ASFV 2.0 PCR Kit (INDICAL Bioscience, Leipzig, Germany), following the manufacturer’s protocol.

### 2.2. Library Preparation for Targeted Tiled Amplicon Sequencing

For targeted tiled amplicon sequencing, the coding-complete genomic region was amplified directly from the extracts following a tiling amplicon long-read sequencing protocol [21] with modifications. Instead of combining the primers into pools, the primer pairs were used individually to detect primer dropouts and to ensure successful amplification of all pairs prior to library preparation and sequencing. Alternate primers were designed based on the previous coding-complete genome sequence we obtained [20] for persistent primer dropouts. Sequences from up to 100 bp upstream of the forward primer and 100 bp downstream of the reverse primer (from low-performing primer pairs) were obtained and were used in subsequent targeted amplification to fill in the gaps. Alternate primers were assessed using the NCBI Primer-blast tool [25]. The PCR reaction was carried out in a 15 μL reaction volume using 1 μL of DNA (5 to 10 ng/μL), 2x PCRBio VeriFi Hot Start master mix (PCR Biosystems Ltd., London, UK), and 200 nM primers, and the thermocycling conditions were as follows: 95 °C for one min, 40 cycles of 95 °C for 15 s, 15 s annealing at 60 °C, and 4 min and 40 s extension at 72 °C. Final extension was set at 72 °C for 10 min. Amplicons were confirmed visually by agarose gel electrophoresis (0.8% *w*/*v* agarose, 3 V per cm, for up to 1 h).

After PCR, the desired amplicons per sample were pooled and purified using a QIAquick PCR Purification Kit (QIAGEN, Venlo, The Netherlands). The purified pooled amplicons were quantified using the Qubit 1x dsDNA HS Assay Kit (Thermo Fisher Scientific, Waltham, MA, USA). A total of 200 fmol amplicon DNA per sample was used as input for the library preparation following the amplicons by ligation (SQK-LSK109) protocol for R9.4.1 flow cells (flongle or minION) with native barcoding (EXP-NBD 104 or EXP-NBD 114) expansion (Oxford Nanopore Technologies [ONT], London, UK). Barcoded samples were pooled equimolarly, and final DNA libraries (50 fmol for MinION or 20 fmol for flongle flow cell) were loaded. Pools of four (4) to six (6) barcoded samples were sequenced at a time on a MinION mk1b device, and the reads were obtained after 24 h running in MinKNOW Software (v. 23.11.7; ONT). The genome of Pangasinan A4 strain (2021), on the other hand, was sequenced previously using SQK-RPB-004 [20].

### 2.3. Genome Assembly and Annotation

Raw reads were base called using the super accurate model (Dorado v. 7.3.9; ONT) and were demultiplexed in MinKNOW Software (v. 23.11.7; ONT) with default parameters. Base called reads per sample were combined in one fastq file, which was used as input to a Snakemake pipeline called LILO [21]. After obtaining the output from LILO, base called reads were mapped against the resulting scaffolds to assess the assembly quality using minimap2 [26] (with ‘-ax map-ont’ option). Sniffles2 [27] was used to detect structural variants (SVs) in the assembled genomes with default parameters. For genomes that could not be assembled using LILO, ViralWasm-Consensus [28] was used as an alternate assembler. Genome coverage was determined using bedtools [29] (v. 2.31.1), minimap2 (v. 2.26-r1175) and samtools [30] (v. 1.19.2).

To annotate the assembled genomes, the GenBank format of the coding sequences from ASFV strain Georgia 2007/1 (Accession: NC_044959.2) was obtained from the NCBI Nucleotide database and compared against the assembled genomes with the Genome Annotation Transfer Utility (GATU) tool [31] v.1.0 using default blastn and needle parameters.

### 2.4. Genetic Characterization and Phylogenetic Analysis

Genomes of the field strains were characterized based on sequences of 13 genes and regions; namely the *B646L* gene, *EP402R* for the CD2v serogroup, the central variable region (CVR) of the *B602L* gene (CVR_B602L_) [32], the intergenic region (IGR) between the *I73R* and *I329L* genes (IGR_I73R-I329L_) [33], the IGR between *A179L* and *A137R* (IGR_A179L-A137R_) [34], other regions such as *CP204L* [35], *O174L*, *K145R* [22], *Bt*/*Sj* and *J268L* [23], *ECO2* (the IGR between *I329L* and *I215L* and the partial *I215L* gene) [24], *MGF 505-5R*, and the *MGF 505 9R* and *10R* regions [13]. Furthermore, these were compared to sequences of several related ASFV strains. Additionally, to confirm the *B646L* genotype of the strains, the latest ASFV p72 genotyping tool and biotyping [36,37] were employed.

Representative genomes from various genotypes obtained from NCBI Datasets (https://www.ncbi.nlm.nih.gov/datasets/) were downloaded (accessed on 1 July 2024) and were compared against the assembled genomes (Table S1). The entire genome sequences were aligned using MAFFT [38] (v. 7.520) in Unipro UGENE software [39] (v. 48.1). Default parameters and maximum likelihood trees were constructed in IQTREE [40] (v. 2.3.1) using ModelFinder [41] and ultrafast bootstrap [42] with 1000 replicates. Phylogenetic trees were visualized using TreeViewer [43] (v.2.2.0).

## 3. Results and Discussion

**Figure 1:**
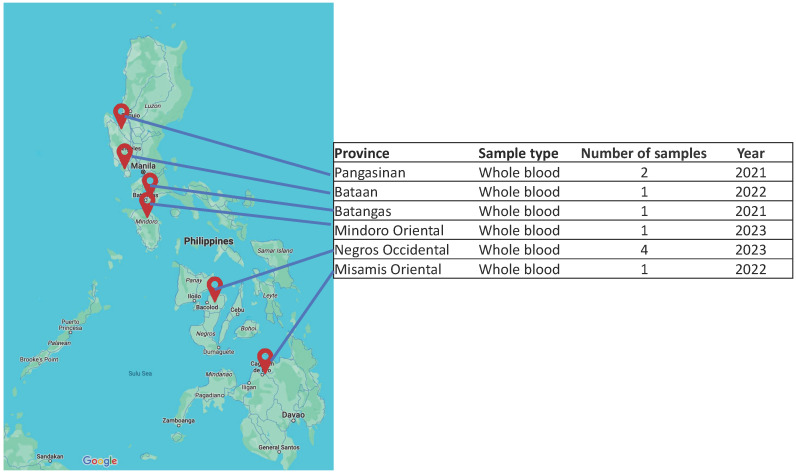
A map of select provinces in the Philippines as sources of ASF-positive samples from 2021 to 2023. Representative samples were obtained from Luzon, Visayas, and Mindanao, Philippines. Map data © 2024 Google.

A total of ten (10) representative ASF-positive whole blood samples from between 2021 and 2023 were obtained from six (6) provinces (Figure 1). Of these samples, the most recent was from Mindoro Oriental, which was a sample from one of the earliest cases in the province in November 2023. Samples from Negros Occidental 2023 cases were obtained from within a few months prior to the outbreak in Mindoro Oriental. The coding-complete genomes of the 10 strains were sequenced and assembled with lengths ranging from 187,609 bp for the Bataan 2022 strain to about 189,500 bp for the rest of the strains (Table 1). Owing to the limitations of the tiled amplicon sequencing method we employed, we were unable to obtain sequences for the terminal inverted repeat regions at both ends of the genome [21]. The assembled genome of ASFV/Philippines/Pangasinan/A4/2021 (ON963982.2) is 192,265 bp in length, which is longer than the assemblies obtained from tiled amplicon sequencing. Shotgun whole-genome sequencing of ASFV-positive blood samples was performed using a different library preparation protocol, resulting in significantly lower mean coverage (21x) compared to tiled amplicon sequencing (1022x up to 5576x). The increased coverage of the tiled amplicons was said to produce more accurate assemblies than shotgun sequencing directly from extracted DNA [21]. All the 10 coding-complete genomes were classified as Biotype 2 together with Georgia 2007/1 and the other 120 genotype II isolates [37].

**Figure 2:**
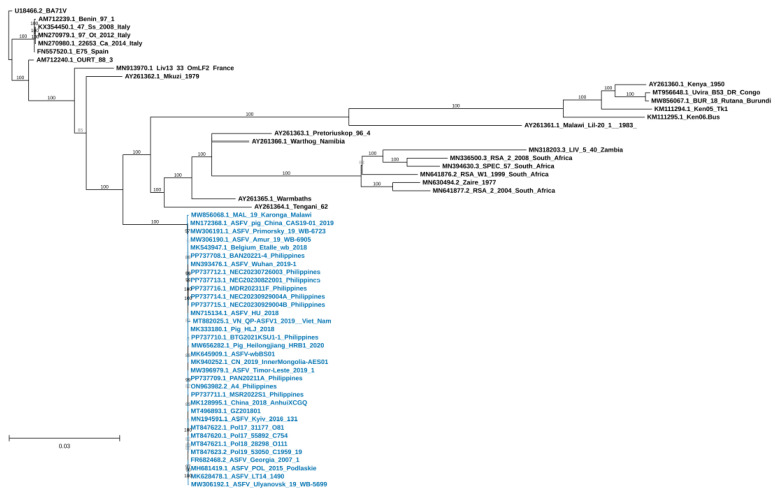
Maximum likelihood consensus tree of select ASFV genomes and the genomes of the Philippine strains inferred using the ultrafast bootstrap implemented in the IQ-TREE software v. 2.3.1 (substitution model: GTR + F + I + R4) with the U18466.2 BA71V genome as the outgroup. The scale bar shows numbers of substitutions per site and bootstrap resampling (1000 iterations) support values are shown at the nodes. ASFV VP72 genotype II genomes are highlighted in blue. Support values lower than 70% are not shown.

A maximum-likelihood phylogenomic tree was constructed to further analyze the genome of ASFVs in the Philippines from the period covered. The tree revealed that the ASFV genomes from the Philippines clustered together with p72 genotype II ASFVs, which include strains detected in Asia and in Europe (Figure 2).

**Figure 3:**
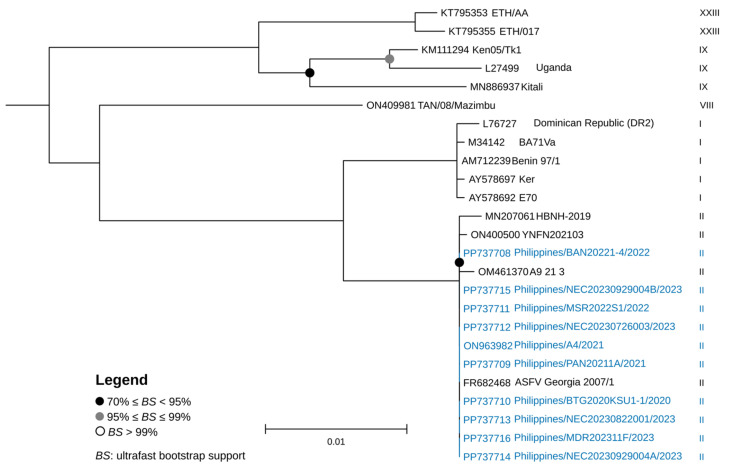
Maximum likelihood tree based on the full-length ASFV p72 (*B646L*) sequence alignment of the recently sequenced nine strains from the Philippines and select publicly available representative ASFV isolates from other genotypes with midpoint roots. Philippine strains are highlighted in blue and the corresponding p72 genotype is indicated. Different genotypes are labeled, respectively. Phylogeny was inferred using the TVM + V + I model in IQTREE following 1000 ultrafast bootstrap iterations. Bootstrap values greater than 70 are indicated at appropriate nodes and the scale bar indicates nucleotide substitutions per site.

**Figure 4:**
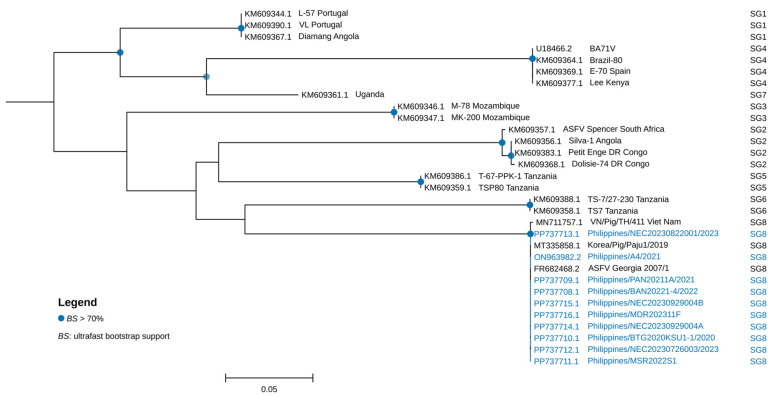
Maximum likelihood tree of full-length ASFV CD2v (*EP402R*) protein sequence alignment of the Philippine strains (highlighted in blue) and representative ASFV strains and corresponding serogroup (SG) is indicated. Phylogeny was inferred using HKY + F + G4 model in IQTREE following 1000 ultrafast bootstrap iterations. Bootstrap values greater than 70 are indicated at appropriate nodes and the scale bar indicates nucleotide substitutions per site.

The complete sequences of genes and genomic regions selected for a multi-gene-based characterization of the Philippine strains were obtained from annotation and through blastn homology searches with Georgia 2007/1 as the reference strain (GenBank Accession No.: FR682468.2). The complete *B646L* and *EP402R* sequences of the 10 Philippine strains obtained from the assembled genomes were 100% identical to the corresponding regions of Georgia 2007/1. All the strains belonged to p72 genotype II and CD2v serogroup 8 (Figure 3 and Figure 4, and Table 2). Among the Eurasian countries, p72 genotype II is the most common circulating genotype [14]. Based on several local ASFV surveillance studies using p72 gene as target, all of the strains so far detected in the Philippines were p72 genotype II [17,44,45]. In South Korea and in many other Asian countries, the most frequently detected p72 genotype II isolates were classified as CD2v serogroup 8 [14,15]. Furthermore, the strains were characterized as belonging to the CVR1 Georgia variant type based on the CVR regions in the *B602L* gene (100% identical to the Georgia 2007/1 strain). CVR1 is also the dominant variant in Asian countries, including South Korea, China, and Vietnam [14,15,46,47].

**Figure 5:**
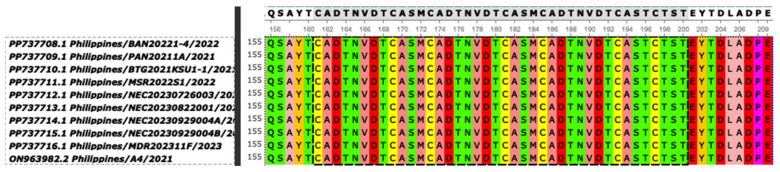
Amino acid sequence alignment of the tetrameric tandem repeat sequences (TRSs) of the central variable region (CVR) of the *B602L* gene of the Philippine strains showing a ‘BNDBNDBNAA’ pattern with 10 repeating patterns. Letters in the CVR sequence represent the TRSs in ASFV strains: A = CAST, CVST, CTST, and CASI; B = CADT, CADI, CTDT, CAGT, and CVDT; N = NVDT, NVGT, and NVDI; D = CASM. Color scheme used was Unipro UGENE Software default for protein alignment.

Amino acid sequence alignment of the tetrameric tandem repeat sequences (TRSs) of the *B602L* CVR of the Philippine strains showed a 10 tandem amino acid repeat sequence pattern (BNDBNDBNAA), which is 100% identical to the CVR of Georgia 2007/1 (Figure 5). There is no report yet of other CVR types (e.g., CVR2, etc.) in the country and in other Asian countries [14].

**Figure 6:**
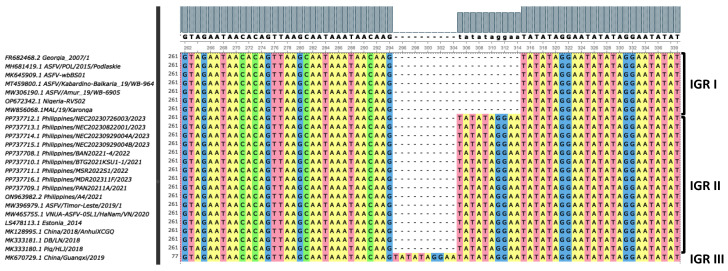
Nucleotide sequence alignment of the intergenic region (IGR) between the *I73R* and *I329L* genes. The Philippine strains belonged to IGR II, with one insertion of 10 nucleotides (GGAATATATA) compared to Georgia 2007/1 (GenBank accession no. FR682468.2). Color scheme used was Unipro UGENE Software default for nucleotide sequence alignment.

The ten local strains were IGR_I73R-I329L_ II variants containing an additional TRS (TATATAGGAA) pattern (Figure 6). Based on the repetition number of TRS, IGR_I73R-I329L_ variants can be classified as IGR I (two copies), IGR II (three copies), IGR III (four copies), and IGR IV (five copies) [33]. In Europe and in Asia, IGR II is the most common genotype [14]. Among the neighboring Asian countries between 2018 and 2023, several IGR I variants were reported in China in 2018, in Vietnam in 2019, and in South Korea in 2019 and in 2023. IGR III variants were likewise detected between 2019 and 2021 in China, Vietnam, and in South Korea. IGR IV variants had been detected in Vietnam since 2021 [14], while in Europe this variant had been detected in Poland and in Germany [22]. IGR_I73R-I329L_ was considered as a genetic marker for the p72 genotype II intragenotypic strain discrimination and was applied in source tracking and tracing of ASFV strains in Eastern Europe [22,33].

**Figure 7:**
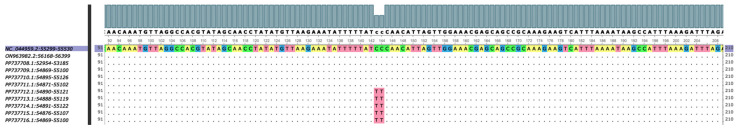
Analysis of IGR_A179L-A137R_ of the 10 ASFV strains from select provinces in the Philippines from outbreaks between 2021 and 2023. Positions 143 and 144 from the start of the IGR of strains from Negros Occidental (PP737712-PP737715) and from Mindoro Oriental (PP737716) had C-to-T substitution. The reference strain (Georgia 2007/1, GenBank Acc. No.: NC_044959) is highlighted. Color scheme used was Unipro UGENE Software default for nucleotide sequence alignment.

For IGR_A179L-A137R_, all 10 strains had the same number of TRSs (two repetitions of ‘GATACAATTGT’) as the Georgia 2007/1 strain. There was no deletion detected but a C-to-T substitution was present at the 143rd and 144th positions in all four (4) Negros Occidental strains and in the Mindoro Oriental strain (Figure 7). Interestingly, these strains were all from 2023 outbreaks and were reported just several weeks apart. It is possible that these strains came from a common source.

In terms of IGR_MGF 505 9R/10R_ analysis (Table 2), all 10 strains were identical to Georgia 2007/1 and were classified as MGF-1 based on the insertion of a 17 nt TRS (GATAGTAGTTCAGTTAA) [48]. Recent expansions of MGF variants were based on the number and type of TRS found near the *9R* and *10R* genes as variations of the 17 nt TRSs ‘AGTAGTTCAGTTAAGAT’ and ‘AGTTCATTTAAGTCAAT’, respectively. Among the IGR_MGF 505 9R/10R_ variants, MGF-1 (with ABBCD__EFGHHH pattern) is the largest group, comprising almost 90% of strains from all sampled countries in one report. Other IGR_MGF 505 9R/10R_ variants (MGF-2 to MGF-8) have been detected in European countries such as Russia, Romania, Lithuania, Latvia, and Poland [24].

The *O174L* gene can also be used in tandem with other genes or regions in strain tracing and source tracking [24]. Variants of this gene are divided into three types: variant I, which is 100% identical to Georgia 2007/1; variant I with SNP; and variant II, with a 14 nt TRS (CAGTAGTGATTTT) insertion. All the 10 Philippine strains belong to *O174L* variant I. Furthermore, *K145R* and *MGF 505-5R* genotyping showed that all the Philippine strains are 100% identical to Georgia 2007/1 and are therefore considered to be variant I (Table 2). Variant II would have G38332A SNP in *MGF 505-5R* [22]. Sequence analysis of the full *K145R* gene from isolates from Kaliningrad, Russia, revealed two SNPs occurring together. One SNP (C>T at position 291) was unique to Kaliningrad isolates and was defined as cluster K145R-III [22,49]. In addition, the other SNP (C>A at position 434) matched a previously identified mutation in Poland and Germany, associated with cluster K145R-II from the EU [18,22].

The region spanning IGR_I329L-I215L_ and the partial *I215L* gene, which is named ECO2, has been used in ASFV strain tracking in Eastern Europe [24]. ECO2 variants can be grouped into four types: the ECO2-I variant is 100% identical to Georgia 2007/1, while the ECO2-II, ECO2-III, and ECOII-IV variants have SNPs at the 62nd position in the *I215L* gene region, as A498G and G446A, respectively. All 10 Philippine strains were ECO2-I variants (Table 2). In Europe and in Asia, most of the ASFV strains sampled were ECO2-I variants, while several ECO2-III and ECO2-IV variants have been detected recently in China [24].

The intergenic *Bt*/*Sj* region of all 10 Philippine strains was identical to Georgia 2007/1, while the *J268L* and *CP204L* genes of all but the Batangas 2021 strain (Philippines/BTG2021KSU1-1/2021) were 100% homologous to the reference strain (Table 2). The Batangas 2021 strain has G-to-A substitution at the 144th position of the *J268L* gene and an A-to-G substitution at the 210th position of the *CP204L* gene relative to the Georgia 2007/1 sequence.

**Figure 8:**
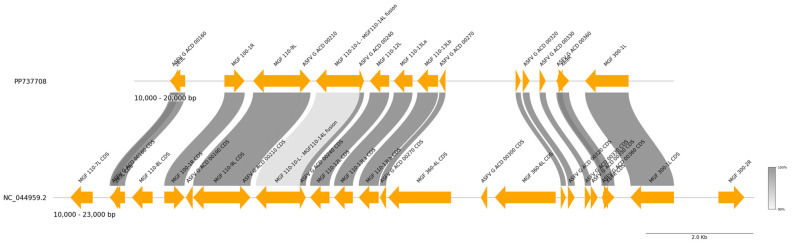
Genome alignment of the Bataan 2022 strain (PP737708) and Georgia 2007/1 (NC_044959.2), showing the genes affected by a 1.9 kb deletion corresponding to the 17,000th and 18,939th positions of the reference Georgia 2007/1 genome. The *MGF 360-4L*, *ASFV G ACD 00300*, and *MGF 360-6L* genes of the Bataan 2022 strain were affected.

The genomes of the 10 Philippine strains were also analyzed for the presence of structural variants (short or long indels). A 1.9 kb deletion was detected in the 5′-end of the genome of the Bataan 2022 strain (Philippines/BAN20221-4/2022), which corresponded to the region between the 17,000th and 18,939th positions of the Georgia 2007/1 genome (Figure 8). The deletion affected the *MGF 360-6L*, *MGF 360-4L*, and *ASFV G ACD 00300* genes.

Members of the *MGF 360* and *MGF 530* variants were observed to be important in virus cell tropism and might be required for efficient virus replication in macrophages [50,51]. In the field isolate OURT88/3 and in the tissue culture-adapted BA71V isolates, deletion of six members of *MGF 360* and up to two members of *MGF 530* resulted in attenuation [52]. On the other hand, the function or role of the *ASFV G ACD 00300* gene is yet to be elucidated. The animal source of the samples from Bataan province showed acute clinical symptoms, suggesting that the strain that infected the animals was not attenuated, despite having a deletion that affected *MGF 360-6L* and *MGF 360-4L*.

The analysis of ASFV whole-genome sequences of strains remains to be the most accurate method for tracing the source of the virus and in understanding its spatiotemporal evolution. However, this method is not without its limitations as it can be time-consuming and is limited by the availability of comparable strain data. Furthermore, high sequence homology among genotype II strains of ASFV, while beneficial for overall genome analysis, can limit the discriminatory power of full genome phylogenetics in resolving closely related outbreaks, as it can make it difficult to identify subtle genetic differences between strains. An alternative is to explore methods, such as focusing on specific regions of the genome with higher mutation rates or using bioinformatics tools to identify subtle differences.

Our in-house tiling amplicon long-read sequencing approach allowed us to obtain coding-complete ASFV genomes that can be subjected to multi-gene-based characterization. Analyzing select gene markers allows for source tracking, strain tracing, and variant detection, and enables differentiation between ASFV strains in order to better understand their introduction and spread. Despite these advantages, care must be taken in interpreting the results of using the current method, as the genetic profiles that can be obtained are not directly related to virulence (except the *CD2v* gene). Through multi-gene-based characterization and whole-genome sequencing, recombinant variants of ASFV have been detected and reported in countries like Russia [53], China [54], and Vietnam [55,56,57]. These strains are significant because they are recombinants of p72 genotypes I and II, with genotype II being previously endemic in the region. In the case of Vietnam, the findings suggested the possibility of multiple independent introductions of these recombinant strains potentially originating from neighboring countries and raise concerns about the effectiveness of current vaccines designed for genotype II ASFV.

The circulating genotype II ASFV strains in the Philippines have already shown evidence of evolution into several variants depending on the genetic markers analyzed. However, given the limited number of samples that were sequenced, no definitive association could be established yet between genetic profiles, geographic origin, and timing of the outbreak among the local strains sequenced, except for the strains from outbreaks that occurred in Negros Occidental and Mindoro provinces during the third and fourth quarter of 2023. These strains showed the same genetic profiles among the markers used in this study.

These findings on strains from select provinces in the country are timely, especially now that the Philippines has authorized the use of modified live ASFV vaccines, as detection of recombinants and variants could complicate ongoing ASFV control measures in the country. Overall, our findings emphasize the ongoing evolution of ASFV, the importance of continuous surveillance for understanding its changing genetic landscape, and the potential implications for diagnostic tools and vaccine development in the country should recombinants or variants arise. Future studies should focus on understanding various ASFV subgroups that could already be present in the Philippines through analysis of additional genetic markers and generation of more whole-genome sequences of strains from other outbreaks.

## Figures and Tables

**Table 1:** Genome sequencing data summary and Cq values of the ten (10) ASFV strains from select provinces in the Philippines.

Strain	Province	Cq Value	Assembly Length (bp)	%GC	Mean Coverage	Predicted ORFs	NCBI Accession No.
ASFV Philippines/BAN20221-4/2022	Bataan	17.6	187,609	38.5	4729x	183	PP737708
ASFV Philippines/PAN20211A/2021	Pangasinan	17.9	189,514	38.4	3183x	187	PP737709
ASFV Philippines/BTG2021KSU1-1/2021	Batangas	20.2	189,540	38.4	4985x	184	PP737710
ASFV Philippines/MSR2022S1/2022	Misamis Oriental	13	189,514	38.4	5576x	175	PP737711
ASFV Philippines/NEC20230726003/2023	Negros Occidental	19.2	189,537	38.4	5092x	188	PP737712
ASFV Philippines/NEC20230822001/2023	Negros Occidental	18.7	189,528	38.4	2673x	188	PP737713
ASFV Philippines/NEC20230929004A/2023	Negros Occidental	19.9	189,539	38.4	2905x	186	PP737714
ASFV Philippines/NEC20230929004B/2023	Negros Occidental	20.3	189,519	38.4	3283x	187	PP737715
ASFV Philippines/MDR202311F/2023	Mindoro Oriental	19.1	189,501	38.4	1022x	187	PP737716
ASFV Philippines/Pangasinan/A4/2021	Pangasinan	21	192,265	38.3	21x	187	ON963982.2

**Table 2:** Comparison of genome features of the 10 ASFV strains from various pig farms in the Philippines between 2021 and 2023 using 13 genes and regions.

Strain	p72 Genotype	CD2v Serogroup	CVR	IGR_I73R-I329L_	IGR_A179L-A137R_	IGR_MGF 505 9R/10R_	ECO2	O174L	K145R	MGF 505-5R	Bt/Sj	CP204L	J268L
Philippines/BAN20221-4/2022	II	8	CVR1	II	No deletion	MGF-1	ECO2-I	I-with deletion	I	I	100%	100%	100%
Philippines/PAN20211A/2021	II	8	CVR1	II	No deletion	MGF-1	ECO2-I	I-with deletion	I	I	100%	100%	100%
Philippines/BTG2021KSU1-1/2021	II	8	CVR1	II	No deletion	MGF-1	ECO2-I	I-with deletion	I	I	100%	One base substitution (A-to-G)	One base substitution (G-to-A)
Philippines/MSR2022S1/2022	II	8	CVR1	II	No deletion	MGF-1	ECO2-I	I-with deletion	I	I	100%	100%	100%
Philippines/NEC20230726003/2023	II	8	CVR1	II	No deletion; with SNPs	MGF-1	ECO2-I	I-with deletion	I	I	100%	100%	100%
Philippines/NEC20230822001/2023	II	8	CVR1	II	No deletion; with SNPs	MGF-1	ECO2-I	I-with deletion	I	I	100%	100%	100%
Philippines/NEC20230929004A/2023	II	8	CVR1	II	No deletion; with SNPs	MGF-1	ECO2-I	I-with deletion	I	I	100%	100%	100%
Philippines/NEC20230929004B/2023	II	8	CVR1	II	No deletion; with SNPs	MGF-1	ECO2-I	I-with deletion	I	I	100%	100%	100%
Philippines/MDR202311F/2023	II	8	CVR1	II	No deletion; with SNPs	MGF-1	ECO2-I	I-with deletion	I	I	100%	100%	100%
Philippines/A4/2021	II	8	CVR1	II	No deletion	MGF-1	ECO2-I	I-with deletion	I	I	100%	100%	100%

## Data Availability

Annotated genome sequences have been deposited in the NCBI GenBank under the accession numbers ON963982.2, and PP737708 to PP737716. The data described here are version 1, except ON963982.2.

## Supplementary Materials

The following supporting information can be downloaded at: https://www.mdpi.com/article/10.3390/v17040588/s1, Table S1: List of ASFV genomes retrieved from NCBI Virus database (accessed 1 July 2024) used in the study.
