# Supplementary material for: Genetic Profiles of Ten African Swine Fever Virus Strains from Outbreaks in Select Provinces of Luzon, Visayas, and Mindanao, Philippines, Between 2021 and 2023
Source: Viruses. 2025 Apr 21;17(4):588. doi: 10.3390/v17040588 (PMC12031577; doi:10.3390/v17040588)
Supplement: Supplementary file 1 [file viruses-17-00588-s001.zip › viruses-3552595-supplementary.pdf]

Table S1. List of ASFV genomes retrieved from NCBI Virus database (accessed 01 July 2024) used in the study.

| Accession No. | Isolate Name | Country | Genotype | Year | Genome Length (bp) |
|---|---|---|---|---|---|
| FR682468.2 | Georgia 2007 | Georgia | II | 2007 | 190584 |
| PP737708.1 | BAN20221-4 | Philippines | II | 2022 | 187609 |
| PP737709.1 | PAN20211A | Philippines | II | 2021 | 189514 |
| PP737710.1 | BTG2021KSU1-1 | Philippines | II | 2021 | 189540 |
| PP737711.1 | MSR2022S1 | Philippines | II | 2022 | 189514 |
| PP737712.1 | NEC20230726003 | Philippines | II | 2023 | 189537 |
| PP737713.1 | NEC20230822001 | Philippines | II | 2023 | 189528 |
| PP737714.1 | NEC20230929004A | Philippines | II | 2023 | 189539 |
| PP737715.1 | NEC20230929004B | Philippines | II | 2023 | 189519 |
| PP737716.1 | MDR202311F | Philippines | II | 2023 | 189501 |
| ON963982.2 | A4 | Philippines | II | 2021 | 192265 |
| MH681419.2 | ASFV/POL/2015/Podlaskie | Poland | II | 2015 | 189404 |
| MK128995.1 | 2018/AnhuiXCGQ | China | II | 2018 | 189393 |
| MK333180.1 | HLJ/2018 | China | II | 2018 | 189404 |
| MK543947.1 | Etalle_wb_2018 | Belgium | II | 2018 | 190202 |
| MK628478.1 | ASFV/LT14/1490 | Lithuania | II | 2014 | 189399 |
| MK645909.1 | ASFV-wbBS01 | China | II | 2018 | 189394 |
| MK940252.1 | CN/2019/InnerMongolia-AES01 | China | II | 2019 | 189403 |
| MN172368.1 | China CAS19-01 | China | II | 2019 | 189405 |
| MN194591.1 | ASFV/Kyiv/2016/131 | Ukraine | II | 2016 | 191911 |
| MN393476.1 | ASFV Wuhan 2019-1 | China | II | 2019 | 190576 |
| MN715134.1 | HU/2018 | Hungary | II | 2018 | 190601 |
| MT496893.1 | GZ201801 | China | II | 2018 | 189393 |
| MT847620.1 | Pol17/55892/C754 | Poland | II | 2019 | 189414 |
| MT847621.1 | Pol18/28298/O111 | Poland | II | 2019 | 189409 |
| MT847622.1 | Pol17/31177/O81 | Poland | II | 2019 | 189422 |
| MT847623.2 | Pol19/53050 C1959 | Poland | II | 2019 | 189413 |
| MT882025.1 | VN/QP-ASFV1(2019) Viet | Viet Nam | II | 2019 | 189081 |
| MW306190.1 | ASFV/Amur 19/WB-6905 | Russia | II | 2019 | 189248 |
| MW306191.1 | Primorsky 19 WB-6723 | Russia | II | 2019 | 189256 |
| MW306192.1 | Ulyanovsk 19 WB-5699 | Russia | II | 2019 | 189263 |
| MW396979.1 | ASFV/Timor-Leste/2019/1 | Timor-Leste | II | 2019 | 192237 |
| MW656282.1 | Pig/Heilongjiang/HRB1/2020 | China | II | 2020 | 189355 |

| | | | | | |
|---|---|---|---|---|---|
| MW856068.1 | MAL/19/Karonga | Malawi | II | 2019 | 183325 |
| AM712239.1 | Benin 97/1 | Benin | I | 1997 | 182284 |
| AM712240.1 | OURT_88/3 | Portugal | I | 1988 | 171719 |
| AY261360.1 | Kenya_1950 | Kenya | X | 1950 | 193886 |
| AY261361.1 | Malawi Lil-20 1983 | Malawi | I | 1983 | 187612 |
| AY261362.1 | Mkuzi_1979 | South Africa | I | 1979 | 192714 |
| AY261363.1 | Pretoriuskop 96/4 | South Africa | XX | 1996 | 190324 |
| AY261364.1 | Tengani 62 | Malawi | V | 1962 | 185689 |
| AY261365.1 | Warmbaths | South Africa | III | 1980 | 190773 |
| AY261366.1 | Warthog Namibia | Namibia | III | 1980 | 186528 |
| FN557520.1 | E75 Spain | Spain | I | 1975 | 181187 |
| KM111294.1 | Ken05 Tk1 | Kenya | X | 2005 | 191058 |
| KM111295.1 | Ken06.Bus | Kenya | IX | 2006 | 184368 |
| KX354450.1 | 47/Ss/2008 | Italy | I | 2008 | 184638 |
| MN270979.1 | 97/Ot/2012 | Italy | I | 2012 | 184206 |
| MN270980.1 | 22653/Ca/2014 | Italy | I | 2014 | 181869 |
| MN318203.3 | LIV 5 40 Zambia | Zambia | I | 1983 | 183292 |
| MN336500.3 | RSA 2_2008 South Africa | South Africa | II | 2008 | 190066 |
| MN394630.3 | SPEC 57 South Africa | South Africa | III | 1985 | 188458 |
| MN630494.2 | Zaire 1977 | Zaire | I | 1977 | 184820 |
| MN641876.2 | RSA W1_1999 South | South Africa | IV | 1999 | 187621 |
| MN641877.2 | RSA 2_2004 South Africa | South Africa | XX | 2004 | 189903 |
| MN913970.1 | OmLF2 | France | I | 2017 | 188277 |
| MT956648.1 | Uvira B53 | DR Congo | X | 2019 | 180916 |
| MW856067.1 | BUR_18 Rutana | Burundi | X | 2018 | 176564 |
| U18466.2 | BA71V | Spain | I | 1971 | 170101 |
